# Effects of social organization and elevation on spatial genetic structure in a montane ant

**DOI:** 10.1002/ece3.8813

**Published:** 2022-05-15

**Authors:** Amaranta Fontcuberta, Martin Kapun, Patrick Tran Van, Jessica Purcell, Michel Chapuisat

**Affiliations:** ^1^ 27213 Department of Ecology and Evolution University of Lausanne Lausanne Switzerland; ^2^ 27271 Center for Anatomy and Cell Biology Department of Cell and Developmental Biology Medical University of Vienna Vienna Austria; ^3^ Natural History Museum of Vienna Vienna Austria; ^4^ Department of Entomology University of California Riverside California USA

**Keywords:** dispersal, landscape genetics, mountain–valley model, population genetics, social polymorphism

## Abstract

Studying patterns of population structure across the landscape sheds light on dispersal and demographic processes, which helps to inform conservation decisions. Here, we study how social organization and landscape factors affect spatial patterns of genetic differentiation in an ant species living in mountainous regions. Using genome‐wide SNP markers, we assess population structure in the Alpine silver ant, *Formica selysi*. This species has two social forms controlled by a supergene. The monogyne form has one queen per colony, while the polygyne form has multiple queens per colony. The two social forms co‐occur in the same populations. For both social forms, we found a strong pattern of isolation‐by‐distance across the Alps. Within regions, genetic differentiation between populations was weaker for the monogyne form than for the polygyne form. We suggest that this pattern is due to higher dispersal and effective population sizes in the monogyne form. In addition, we found stronger isolation‐by‐distance and lower genetic diversity in high elevation populations, compared to lowland populations, suggesting that gene flow between *F*. *selysi* populations in the Alps occurs mostly through riparian corridors along lowland valleys. Overall, this survey highlights the need to consider intraspecific polymorphisms when assessing population connectivity and calls for special attention to the conservation of lowland habitats in mountain regions.

## Graphical abstract

We studied how social organization and landscape factors affect spatial patterns of genetic differentiation in an ant species living in mountainous regions. Genetic differentiation between populations was weaker for the monogyne form than for the polygyne form. In addition, we found stronger isolation‐by‐distance and lower genetic diversity in high elevation populations, compared to lowland populations, suggesting that gene flow between *Formica selysi* populations in the Alps occurs mostly through riparian corridors along lowland valleys.

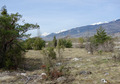

## 1 INTRODUCTION

Sociality has profound effects on population processes (Szathmary & Maynard Smith, 1995; Wilson, 1992). Because few individuals reproduce in each group, eusocial insects (ants, bees, wasps, and termites) have comparatively smaller effective population sizes than solitary insects (Pamilo & Crozier, 1997), which translates into lower genetic diversity within populations, and potentially higher inbreeding (Chapman & Bourke, 2001; Hedrick & Parker, 1997). Social insects often form sessile and perennial colonies, and philopatry of reproductive individuals is common (Le Galliard et al., 2005; Seppä, 2008), so that local population genetic structure is expected (Ross, 2001). Together, small effective population size, low diversity, and strong population structure reduce selection efficiency and adaptive capacity (Romiguier et al., 2014; Settepani et al., 2016; Weyna & Romiguier, 2021). Therefore, some aspects of sociality may hamper the capacity of social insects to respond to rapid environmental change, presenting an added challenge for their conservation (Chapman & Bourke, 2001; Fisher et al., 2019; Seppä, 2008).

The ability of social insects to disperse and cope with environmental change depends on their social organization. Ant colonies can have a single queen (= “monogyne”) or multiple queens (= “polygyne”). The monogyne and polygyne social forms generally differ in several traits, including colony size and lifespan, sex allocation, dispersal, and colony founding strategy (Keller, 1993). Across species, queens of the monogyne social form disperse on the wing and establish novel colonies independently. In contrast, queens of the polygyne social form have the additional options of staying in their natal nests and establishing new polygyne colonies by dispersing on foot with workers (“colony budding,” Bourke & Franks, 1995). Because of higher long‐range dispersal, population genetic structure is generally weaker in monogyne species, compared to polygyne species (e.g., Chapuisat et al., 1997; Ross, 2001; Seppä & Pamilo, 1995). This pattern has also been documented between monogyne and polygyne populations of polymorphic species (e.g., Huszár et al., 2014; Ross & Shoemaker, 1997; Sundström et al., 2005). Yet, when social forms are allopatric, the effects of social organization, geography and ecology are confounded. Socially polymorphic species in which monogyne and polygyne colonies occur in sympatry offer the opportunity to study the direct effects of social organization on dispersal and population genetic structure.

Several landscape factors tend to restrict gene flow and lead to population structure. First, gene flow may be constrained by geographical distance, causing distant populations to diverge through drift (“isolation‐by‐distance,” Wright, 1943). The process is exacerbated by barriers to movement, such as water bodies, high mountains, or urbanized areas. Second, populations may experience ecological isolation, leading to divergent selection and local adaptation (“isolation‐by‐environment,” Wang & Bradburd, 2014). These factors, alone or in combination, act at multiple spatial scales, and may lead to complex population genetic patterns in heterogenous landscapes (Cushman et al., 2006; Meirmans, 2012). Mountains encompass a great range of elevation, climate and ecosystems within small regions. Thus, mountain regions are prime areas to investigate how social organization and landscape factors interact in shaping dispersal and population structure.

Here, we study the population genetic structure of a montane ant species, *Formica selysi*. This socially polymorphic ant is a pioneer species colonizing floodplains along mountain rivers (Chapuisat et al., 2004; Lude et al., 1999; Zahnd et al., 2021). Natural floodplains are among the most diverse ecosystems on earth, but are highly threatened: up to 90% of natural European floodplains have disappeared as a result of human activity (Tockner & Stanford, 2002). Although *F*. *selysi* can be locally common (Zahnd et al., 2021), it is considered a threatened species in certain parts of the European Alps (Glaser, 2005).

Most well‐sampled populations of *F*. *selysi* have both monogyne and polygyne colonies (Chapuisat et al., 2004; Purcell et al., 2015). Colony social organization is controlled by a large supergene with two haplotypes, *M* and *P* (previously called *Sm* and *Sp*; Purcell et al., 2014). Queens and workers in monogyne colonies are homozygous for the *M* haplotype, whereas queens and workers in polygyne colonies are homozygous for the *P* haplotype or heterozygous (*MP* genotype; Purcell et al., 2014; Avril et al., 2019). Outside of the supergene, there is little genetic differentiation between social forms (Chapuisat et al., 2004; Purcell et al., 2014; Purcell & Chapuisat, 2013), suggesting extensive gene flow.

The monogyne and polygyne social forms of *F*. *selysi* differ in a suite of traits, including sex allocation, dispersal, and mode of colony founding. Monogyne colonies produce 90% of the alate females (the future queens) dispersing by flight (Fontcuberta et al., 2021). These females of monogyne origin are larger and more successful at independent colony founding than females produced by polygyne colonies (De Gasperin et al., 2020; Reber et al., 2010; Rosset & Chapuisat, 2007). Some females of polygyne origin also disperse by flight and found colonies independently (Blacher et al., 2021; De Gasperin et al., 2020; Fontcuberta et al., 2021; Reber et al., 2010; Rosset & Chapuisat, 2006). Females from polygyne colonies tend to mate with slightly related males (Avril et al., 2019), which suggests that some of the polygyne females mate inside or close to their natal nest and forgo dispersal.

Restricted dispersal of polygyne females is expected to result in stronger population genetic structure and isolation‐by‐distance in the polygyne social form, compared to the monogyne form (Ross, 2001; Sundström et al., 2005). However, male‐mediated gene flow within and between social forms might erode population genetic structure (Avril et al., 2019). Previous studies did not detect strong differences between *F*. *selysi* social forms in the degree of isolation‐by‐distance among colonies within populations (Avril et al., 2019; Chapuisat et al., 2004). Whether genetic structure differs between social forms at a larger geographical scale has not been investigated so far.

A previous genetic survey of several populations in the Alps revealed that large river drainage basins have a strong influence on spatial genetic differentiation in *F*. *selysi* (Purcell et al., 2015). Little genetic differentiation was detected between populations within mountain valleys, suggesting high gene flow along elevation gradients. Dispersal success depends on the ability to cross geographical barriers and availability of suitable habitat within flying distance (Hakala et al., 2019). The ability of *F*. *selysi* to fly over long distances and cross mountain ridges is unknown. Its habitats consist of gravel and sandy floodplains along rivers, which are rare in steep mountains valleys and become more and more fragmented with increasing active management of water courses (Ballinger et al., 2007). Thus, more research is needed to understand how this riverine ant species disperses and colonizes its discontinuous mountain habitats, and how elevation affects population connectivity.

We used genome‐wide ddRAD‐seq markers to infer the population genetic structure of *F*. *selysi* across several valleys in the European Alps. The sampling scheme covers a large portion of the species range. We sampled populations in three well‐separated geographical regions belonging to two drainage basins (Rhône and Rhine, Purcell et al., 2015) and comprising strong elevation contrasts in independent valleys. Our goals were first, to identify landscape factors affecting gene flow in mountains; and second, to investigate how intraspecific variation in social organization affects patterns of population structure. Overall, this study sheds light on factors affecting population connectivity and dispersal in social insects, which can prove valuable for conservation management.

## 2 METHODS

### 2.1 Sampling and genotyping

**FIGURE 1:**
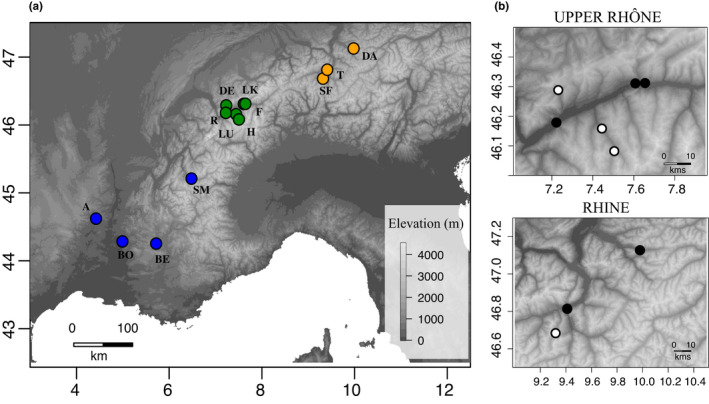
Map of sampling localities (=populations). (a) Colors indicate three well‐separated regions, blue: Lower Rhône region (A: Aubenas, BO: Buisson, BE: Bussets, SM: St‐Michel), green: Upper Rhône region (F: Finges, LK: Leuk, R: Riddes, LU: Luette, H: Les Haudères, DE: Derborence), orange: Rhine region (T: Tamins, SF: Safien, DA: Dalaas). (b) Zoom of the localities in the Upper Rhône and Rhine regions. White circles are highland populations (>1,000 m), and black circles are lowland populations (<1,000 m). Axes show the longitude and latitude. The grey tones in the background maps show elevation, based on SRTM elevation raster data with 30 m resolution

*Formica selysi* lives in riverine ecosystems throughout the European Alps and the Pyrenees mountains (Seifert, 2002). We sampled workers in 152 colonies from 13 localities ranging from 180 m to 1,450 m in elevation (1–32 colonies per locality, Table A1). In each locality monogyne and/or polygyne colonies were sampled within a 1 km^2^ area (Table A1). The sampling localities were situated along the Rhine River or tributaries (3 localities, east Switzerland and west Austria), along the Upper Rhône River or tributaries (6 localities, west Switzerland), and along tributaries of the Lower Rhône River (4 localities, France; Figure 1, Table A1). Each locality represents a separate population.

We genotyped one worker per colony. We extracted DNA from the head and thorax of each worker using the Qiagen DNeasy Blood and Tissue kit, following the protocol for insect tissue. We obtained double‐digest RAD sequence data by following the ddRAD‐seq protocol described in Brelsford et al. (2016). In brief, we digested genomic DNA using restriction enzymes *EcoRI* and *MseI*, ligated inline barcoded adapters, removed DNA fragments shorter than 250 bp using AMPure magnetic beads, carried out PCR amplification of each individual in triplicate, during which we added a second unique adapter for each independent plate, and carried out a final size selection on the pooled libraries, to retain sequences in the 400–500 bp range. The resulting libraries were sequenced on the Illumina 2500 Hi Seq platform of the Lausanne Genomic Technologies Facility.

### 2.2 Bioinformatics

Demultiplexing and quality control of raw sequences were done with the *process_radtags* pipeline in STACKS v. 2.2 (Catchen et al., 2013). Clean reads were aligned to an upgraded version of the reference genome of *Formica selysi* (Brelsford et al., 2020, NCBI, GenBank accession number: GCA_009859135.1), using BWA v. 0.7.17 (Li & Durbin, 2009). Single Nucleotide Polymorphisms (SNPs) and genotypes were called with the *ref_map* pipeline in STACKS, using default parameters. The initial consensus output catalogue from the *populations* program contained 628,232 RAD loci, with average length of 84.7 bp and average sample coverage of 28.9x. In total, 323,797 SNPs were retained, distributed across 99,299 polymorphic RAD loci.

Further SNP filtering was done using the VCFtools (Danecek et al., 2011) and the “VcfR” *R* package (Knaus & Grünwald, 2017). Genotypes with quality score lower than 20 and sequencing depth lower than three‐folds were considered missing data. We retained one random polymorphic site per RAD locus, to avoid bias due to linkage disequilibrium. We removed sites with heterozygosity higher than 0.70, to exclude merging paralogous loci (Paris et al., 2017). We only retained SNPs with minor allele frequency higher than 0.01 and mapping to one of the 27 chromosome‐length scaffolds of the reference genome. We further removed individuals with more than 30% of missing data and selected SNPs present in 95% of the individuals retained. The resulting dataset had 13,421 SNPs, of which 923 were on chromosome 3, which contains the non‐recombining social supergene (Purcell et al., 2014), and 12,498 were in the remaining 26 chromosomes.

### 2.3 Determination of social form

**FIGURE A1:**
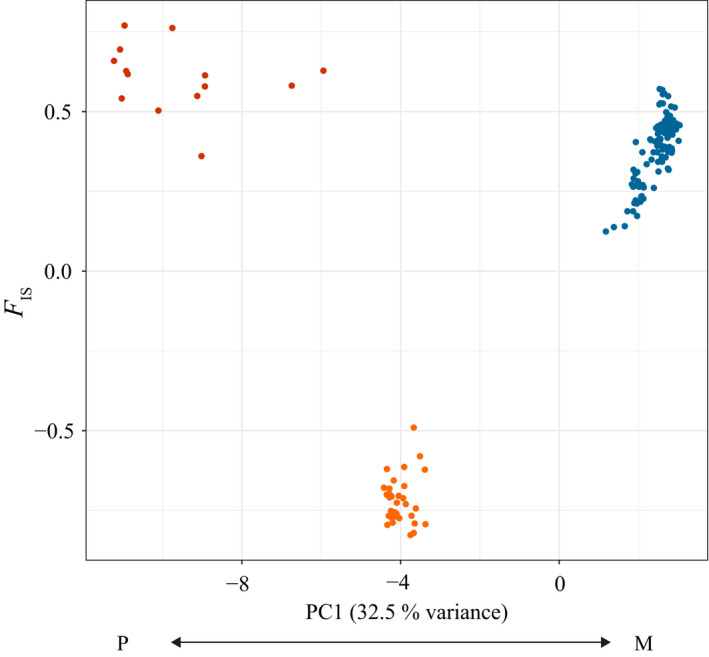
Determination of the social supergene genotype. X‐axis displays the first component (PC1) of a PCA of SNPs on chromosome 3, which contains the social supergene. Y‐axis represents the *F*_IS_ per individual: negative values indicate heterozygous genotypes and positive values indicate homozygous genotypes. The three groups correspond to the social supergene genotypes *PP* (red), *PM* (orange) and *MM* (blue), respectively. *PP* and *PM* individuals belong to the polygyne social form, whereas *MM* individuals belong to the monogyne social form (Avril et al., 2019; Purcell et al., 2014)

We inferred the social form of each individual from their social supergene genotype (Brelsford et al., 2020; Purcell et al., 2014). Individuals in monogyne colonies are homozygous for the *M* haplotype, whereas individuals in polygyne colonies are either homozygous for the *P* haplotype or heterozygous (*MP* genotype; Purcell et al., 2014; Avril et al., 2019). Worker genotypes were perfectly associated with colony queen number across hundreds of individuals from both types of colonies, suggesting that worker drifting between social forms is unlikely (Avril et al., 2019; Fontcuberta et al., 2021; Purcell et al., 2014; Zahnd et al., 2021). To determine the supergene genotype of each individual, we ran a PCA on SNPs in chromosome 3, using the “adegenet” R package (Jombart & Ahmed, 2011). The first component (32.5% of variance) distinguishes the three supergene genotypes. The inbreeding coefficient (*F*_IS_), calculated with VCFtools, distinguishes homozygous from heterozygous individuals (Figure A1). Overall, 106 individuals belonged to the monogyne social form, whereas 46 individuals belonged to the polygyne social form (Table A1). We will refer to them as monogyne and polygyne individuals, respectively.

### 2.4 Population genetic analyses

All analyses were carried out in R v. 2.4.01 (R Core Team, 2020), using the 12,498 SNPs located in chromosomes other than chromosome 3, since the supergene evolves independently from the rest of the genome and including the non‐recombining supergene haplotypes would not reflect population genetic structure. Genetic variation among individuals was investigated by clustering individuals with DAPC (discriminant analysis of principal components; Jombart et al., 2010) based on allele frequencies, using the “adegenet” package. To best identify the number of genetic clusters, we ran K‐means algorithm with the function *find*.*clusters*, with K ranging from 1 to 15, and selected the number of clusters K with the lowest Bayesian information criteria (BIC). We further inferred population genetic structure with hierarchical *F*‐statistics analyses, and obtained 95% confidence intervals (CI) by bootstraping over loci, as implemented in the R package “hierfstat” (Goudet, 2005). The hierarchical levels were regions, populations within regions, and social forms within populations.

We tested for isolation‐by‐distance (IBD) and isolation‐by‐environment (IBE) between pairs of populations, excluding two populations in which fewer than three individuals were sampled (Aubenas and Dalaas, Figure 1, Table A1). Genetic distances between population pairs were calculated with the function *betas* in “hierfstat.” This function uses the Weir and Goudet estimator of *F*_ST_, which is robust to unequal sample sizes and appropriate for SNPs markers with allele dosage information (Weir & Goudet, 2017). We calculated geographical great‐circle distance, elevation distance, and four multivariate environmental distances, namely temperature, precipitation, soil, and vegetation (Table A2). Environmental variables were estimated using raster data from public databases (Table A2). They were scaled and centered to account for differences in magnitude (Lichstein, 2007). The environmental distances were then calculated as euclidian dissimilarities, using the R package “ecodist” (Goslee & Urban, 2007). We used separate Mantel tests to examine the association between genetic distance (*F*_ST_) and each of the other distances. Next, we ran a multiple regression of distance matrices (MRM, Lichstein, 2007) with the genetic distance (*F*_ST_) as response variable and geographical distance, elevation distance and the four environmental distances as predictors. These tests were run in “ecodist,” and the significance of the associations tested with 1,000 permutations.

We investigated if elevation and social organization affected isolation‐by‐distance and population differentiation at a local scale, within regions. For that, we used maximum‐likelihood population‐effects models, which are linear mixed‐effect regression models (LMER) that include a random term to account for correlation of pairwise distances involving a common population (Clarke et al., 2002; Van Strien et al., 2012; Yang, 2004). To test if elevation impacts genetic differentiation between populations, we focused on the upper Rhône and Rhine regions, since they comprise populations close to each other and differing strongly in elevation (Figure 1B, Table A1). We classified populations in two categories: lowland (<1,000 m, range 473–631 m) or highland (>1,000 m, range 1,045–1,455 m). We included *F*_ST_ between each pair of populations as the response variable in a LMER. The geographical distance, elevation category combination (lowland–lowland, lowland–highland, and highland–highland), and interaction between the two factors were included as fixed explanatory factors, while a random term accounted for correlation of pairwise distances. Additionally, we tested for the effect of elevation on genetic diversity. Genetic diversity (*H*s, expected heterozygosity, averaged across loci) within each population was estimated with the “hierfstat” package. We ran a linear model with genetic diversity (*H*s) as response variable and elevation category (lowland or highland) as well as region as explanatory variables.

To test if social organization affects genetic differentiation between populations within regions, we focused on pairs of populations less than 100 km apart. These pairs comprise all populations with three or more individuals, except St. Michel in the Lower Rhône region, which is distant from all other populations (Figure 1, Table A1). We calculated *F*_ST_ between individuals belonging to the monogyne (M) or the polygyne (P) social form in each population, resulting in distances corresponding to three social form combinations between each population pair (M‐M, P‐P, and M‐P). To control for sample size bias, we calculated *F*_ST_ with rarefaction and 1,000 iterations of resampling, taking as sample size the smallest number of individuals belonging to one social form in one population of this pair. For example, for the *F*_ST_ between "Derborence‐M" (*N* = 14) and "Finges‐M" (*N* = 22), we resampled 10 individuals from each of the two groups, corresponding to the smallest sample size for one social form in this population pair, which is "Finges‐P" (*N* = 10; Table A1). We ran a LMER model with pairwise *F*_ST_ as a response variable. We included as fixed explanatory factors the geographical distance between two populations, the social form combination (M‐M, P‐P, or M‐P), and the interaction between the two factors. We also included a random term to account for correlation of pairwise distances.

We checked for normality, homoscedasticity and absence of overdispersion of residuals in all statistical models by visual inspection of plots, as well as tests implemented in the “DHARMa” package (Hartig, 2018). LMER models were ran with the package “lme4” (Bates et al., 2014). ANOVA type III estimates and *p*‐values for the LMER models were obtained using the Kenward Roger approximation with the function *KRmodcomp* in the “pbkrtest” package (Halekoh & Højsgaard, 2014), and with the *drop1* function for the linear model. We performed post‐hoc Tukey tests on estimated marginal means, as implemented in the “emmeans” package (Lenth et al., 2020).

## 3 RESULTS

**FIGURE 2:**
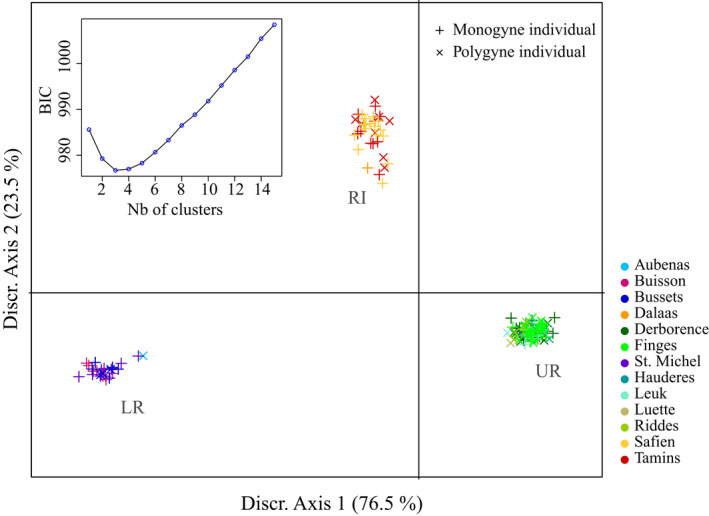
Genetic clustering of individuals by DAPC, with *K* = 3 clusters. Each cross represents a monogyne (+) or polygyne (X) individual, colored by population. LR cluster: individuals from Lower Rhône region; UR cluster: individuals from Upper Rhône region; RI cluster: individuals from Rhine region. *Caption*: Bayesian information criteria for *K* = 1–15, used for determining K. Includes individuals from all populations sampled

The clustering analysis and hierarchical *F*‐statistics revealed high genetic differentiation among regions, moderate differentiation among populations within regions, and low differentiation between social forms within populations (Figure 2). These analyses were based on 12,498 SNPs spanning the entire genome except chromosome 3, which contains the social supergene. Individuals clustered in three geographically concordant genetic groups, distinguishing individuals from east Switzerland and Austria (Rhine region), central Switzerland (Upper Rhône region), and southeast France (Lower Rhône region; Figures 1 and 2). DAPC clustering confirmed that the best number of clusters was *K* = 3, separating the three regions (Figure 2).

In hierarchical *F*‐analyses, differences among regions explained most of the genetic variance across landscape (*F*_region‐total_ = 0.084, 95% CI = [0.082,0.087]). Differentiation among populations within regions was moderate (*F*_population‐region_ = 0.047, 95% CI = [0.046,0.049]), while differentiation between social forms within populations was low (*F*_social form‐population_ = 0.017, 95% CI = [0.015, 0.018]), indicating extensive gene flow between social forms. Overall, individuals clustered by regions, but not by population or social form, at SNPs located outside of the social supergene.

### 3.1 Isolation‐by‐distance and isolation‐by‐environment

**FIGURE 3:**
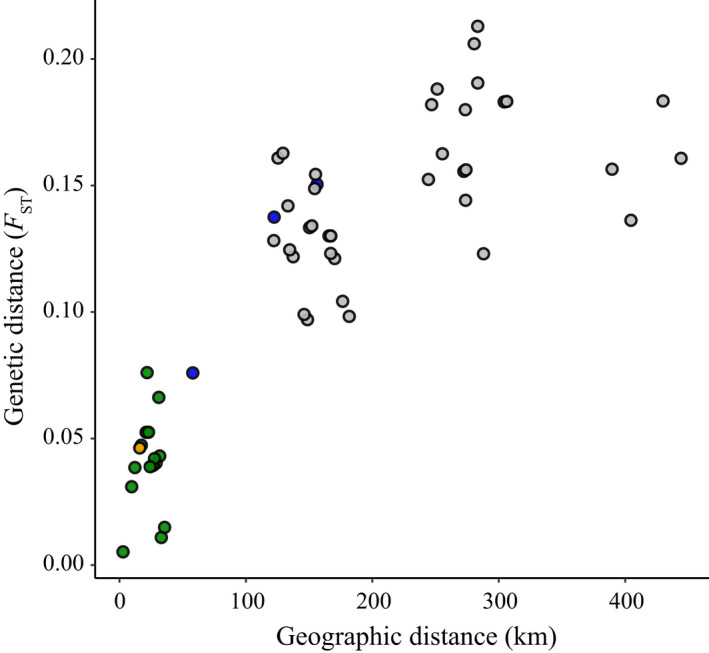
Isolation‐by‐distance. Relation between genetic distance (*F*_ST_) and geographical distance across pairs of populations. Colored dots are population comparisons within regions (blue: Lower Rhône, green: Upper Rhône, and orange: Rhine), and grey dots represent comparisons between populations from different regions. Includes populations BO, BE, and SM in Lower Rhône, all populations in Upper Rhône, and T and S populations in Rhine region (Table A1)

**FIGURE A2:**
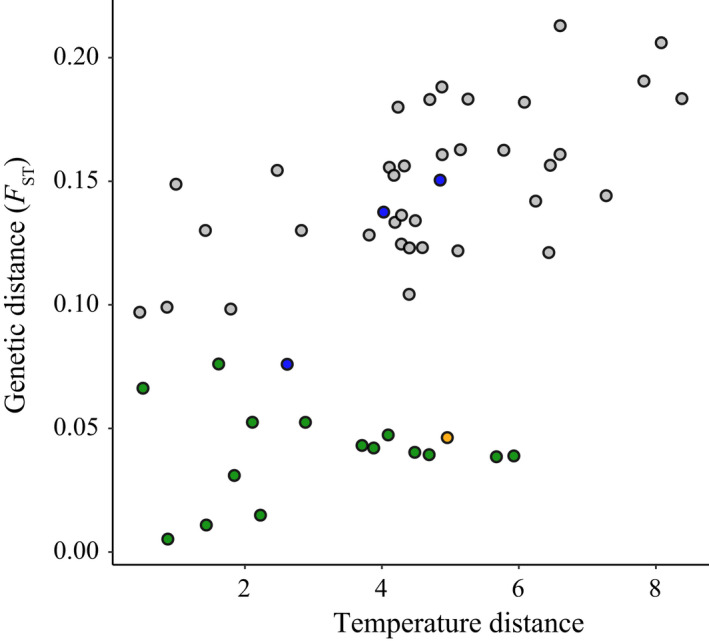
Isolation by environment. Relation between genetic distance (*F*_ST_) and multivariate temperature distance. Coloured dots are population pairs within regions (blue: Lower Rhône, green: Upper Rhône, orange: Rhine), and grey dots represent population pairs from different regions. Includes populations BO, BE and SM in Lower Rhône, all populations in Upper Rhône, T and S populations in Rhine region (Table A1)

There was a very strong pattern of isolation‐by‐distance at a range‐wide scale (Mantel test: *R* = .83, *p* < .001; Figure 3). Genetic distance between populations was also significantly correlated with temperature distance (Mantel test: *R* = .54, *p* < .001, Figure A2), but not with any of the other environmental or elevation distances. In a multiple regression matrix (MRM) that included geography, elevation, and the four environmental distances, only geographical distance was significantly associated with genetic distance (MRM: *R* ^2^ = .72; geography: *p* = .001; elevation: *p* = .77; temperature: *p* = .1; precipitation: *p* = .27; soil: *p* = .61; vegetation: *p* = .84). This suggests that the effect of temperature is due to its correlation with geography, and that geography accounts for most of the genome‐wide genetic differentiation across the range.

### 3.2 Effects of elevation and social organization on population differentiation

**FIGURE 4:**
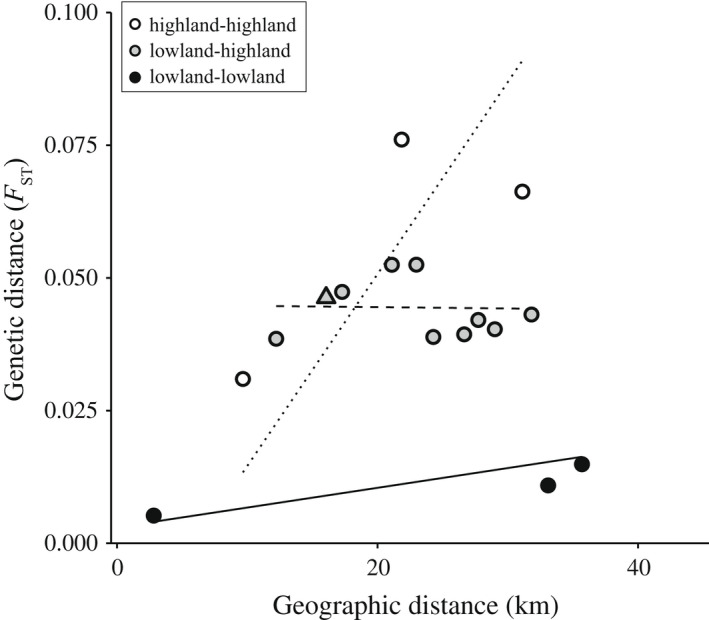
Isolation‐by‐distance according to elevation. Isolation‐by‐distance (genetic distance relative to geographical distance) between pairs of lowland populations (black dots, thick line), pairs of highland populations (white, dotted line) or pairs of lowland and highland populations (grey, dashed line) from the same region (circles: Upper Rhône, triangle: Rhine). Lines represent predicted values from the LMER model, which includes as explanatory variables geographical distance, elevation category, and their interaction. Includes all populations in Upper Rhône region, and populations T and S in Rhine region (Table A1)

**FIGURE A3:**
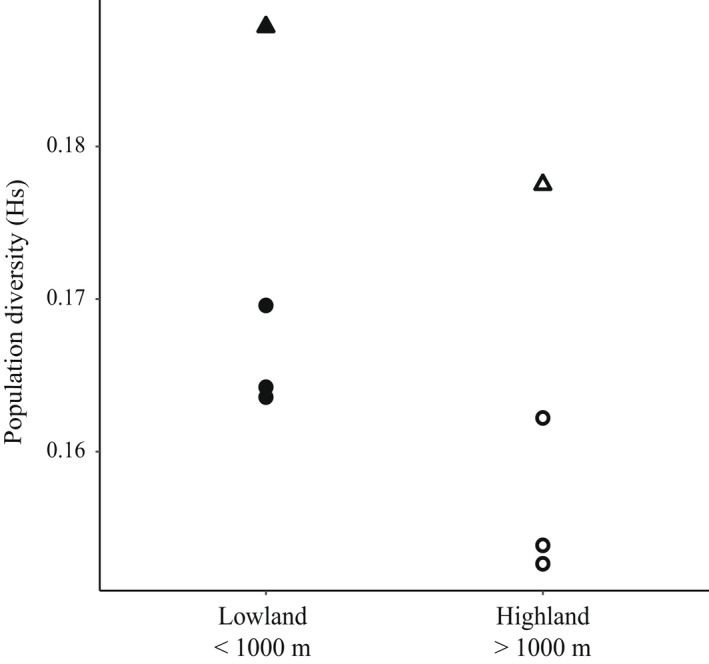
Genetic diversity in lowland (black) and highland (white) populations. Each dot represents a population (circles: Upper Rhône, triangles: Rhine). Includes all populations in Upper Rhône region, and populations T and S in Rhine region (Table A1)

Isolation‐by‐distance between populations within regions varied with elevation. The association between genetic and geographical distance was stronger among highland populations than among lowland populations (LMER, interaction “geographical distance” and “elevation”: *F* = 133.97, df = 2, 3.1, *p* < .001; Figure 4). Genetic distance was higher between pairs of highland populations than between pairs of lowland populations (LMER, “elevation”: *F* = 11.46, df = 2, 6.3, *p* = .008; Tukey post‐hoc test, estimate “lowland–lowland” vs. “highland–highland” = −0.051, SE = 0.01, df = 6.2, *t* = −4.97, *p* = .006), while genetic distances between lowland and highland populations were intermediate (post‐hoc tests, estimate “lowland–lowland” vs. “highland–lowland” = −0.035, SE = 0.0086, df = 4.8, *t* = −4.05, *p* = .024; estimate “highland–highland” vs. “highland–lowland” = −0.016, SE = 0.006, df = 7.3, *t* = −2.56, *p* = .082). Genetic diversity was higher in lowland populations than in highland populations (LM, estimate “elevation” = −0.007, SE = 0.002, *t* = −3.65, *p* = .015; Figure A3). Populations in the Rhine region were genetically more diverse than populations in the Upper Rhône region (estimate “region” = −0.022, SE = 0.003, *t* = −6.9, *p* < .001).

**FIGURE 5:**
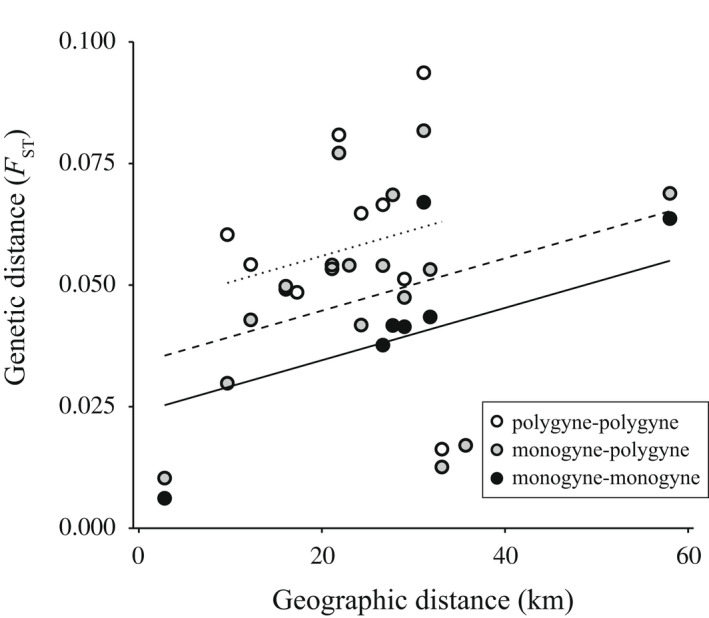
Isolation‐by‐distance according to social organization. Isolation‐by‐distance within and between social forms, across pairs of populations. Each dot represents the distance between a pair of populations, considering only individuals belonging to the monogyne social form (black circles, thick line), only individuals belonging to the polygyne social form (white circles, dotted line), or monogyne individuals in one population and polygyne individuals in the other population (grey circles, dashed line). Lines represent predicted values from the LMER model, which includes as explanatory variables geographical distance and social form. Includes all populations in Upper Rhône region; populations T and S in Rhine region and populations BO and BE in Lower Rhône region (Table A1)

Social organization affected the genetic distance between populations within regions (LMER, “social form”: *F* = 17.28, df = 2, 18.6, *p* < .001; Figure 5). For the same population pairs, genetic distances were higher when considering individuals belonging to the polygyne social form than when considering individuals belonging to the monogyne social form (Tukey post‐hoc test, estimate “P‐P” vs. “M‐M” = −0.021, SE = 0.0037, df = 19, *p* < .001). Distances between monogyne individuals of one population and polygyne individuals of the other were intermediate (post‐hoc tests, estimate “M‐P” vs “P‐P” = −0.010, SE = 0.003, df = 18.7, *p* = .002; estimate “M‐P” vs. “M‐M” = −0.011, SE = 0.003, df = 18.5, *p* = .006). The degree of isolation‐by‐distance did not differ according to the social form considered (interaction “social form” and “geographical distance”: *F* = 1.5, df = 2, 16.9, *p* = .26).

## 4 DISCUSSION

Patterns of population genetic structure depend on landscape structural features, but also on species‐specific traits that determine how organisms respond to geographical constraints (Baguette et al., 2013). In this population genomics survey, we investigated how topographic and environmental factors affect spatial genetic patterns in a montane ant species, and whether these patterns vary between alternative genetically determined social forms within this species. We detected a strong pattern of isolation‐by‐distance at a range‐wide scale, but only moderate genetic structure within regions, especially among lowland populations. Moreover, spatial genetic structure differs between social forms.

Such strong pattern of isolation‐by‐distance between populations (IBD) is uncommon in ants (but see Flucher et al., 2021). In a review of 14 species of the *Formica* genus, Sundström et al. (2005) found IBD at inter‐population scale in only one species. IBD is more common at a local scale, that is, between colonies within populations (reviewed in Johansson et al., 2018; Sundström et al., 2005). Differentiation between populations from distinct regions (pairwise *F*_ST_ = 0.1–0.2) was high compared to measures over similar geographical scales in other ant species.

High population genetic differentiation and isolation‐by‐distance in *F*. *selysi* across the European Alps may be explained by the ecology of this riverine species. Suitable habitats—natural floodplains—tend to be discontinuous along river valleys, which restricts the possibilities of successful colony founding and limits gene flow. Moreover, distant regions might correspond to independent glacial refugia (Purcell et al., 2015; Schmitt, 2009; Trettin et al., 2016). Low connectivity of riverine ecosystems between regions and colonization of regions from distinct sources are not mutually exclusive, and can together account for the strong genetic differentiation detected between distant populations, across the species range.

Intraspecific variation in social organization affected population structure within regions, irrespective of geographical distance. Population differentiation was stronger for the polygyne social form than for the monogyne social form. Previous studies within one large *F*. *selysi* population found that spatial genetic differentiation above the colony level was similar in the two social forms, at a local scale (Avril et al., 2019; Chapuisat et al., 2004). Our new results reveal that social organization affects spatial genetic structure at a larger, inter‐population spatial scale.

Stronger genetic differentiation between polygyne populations than between monogyne ones has been documented in other ant species (Ross & Shoemaker, 1997; Seppä et al., 2004; Seppä & Pamilo, 1995; Sundström et al., 2005). Yet, in these species, polygyne and monogyne colonies occur in geographically separated populations, so that differences in spatial genetic patterns may be explained by other environmental correlates. In *F*. *selysi*, monogyne and polygyne colonies co‐occur within the same locations. Therefore, the association between social form and spatial genetic structure is due to differences in social organization, and not to other correlated geographical effects.

Strong genetic differentiation in the polygyne social form could be caused by restricted female dispersal, recurrent founder effect and/or smaller effective population size. Each of these factors tends to reduce genetic diversity and increase *F*_ST_ (Ross, 2001). In *F*. *selysi*, monogyne colonies produce numerous females that disperse on the wing, while polygyne colonies produce very few females (Fontcuberta et al., 2021; Rosset & Chapuisat, 2006). Moreover, monogyne females are larger (by 59% in dry weight and 2% in head width), more fertile and more successful at independent colony founding, while polygyne females are smaller, less fertile, and more philopatric (Avril et al., 2019; De Gasperin et al., 2020; Fontcuberta et al., 2021; Reber et al., 2010; Rosset & Chapuisat, 2007). Most of the monogyne females (~80%) mate with monogyne males and yield monogyne colonies (Fontcuberta et al., 2021). Thus, females that manage to reach distant populations and establish novel colonies independently are much more likely to belong to the monogyne social form.

Monogyne females mated to monogyne males and producing monogyne colonies are probably the main dispersers and founders across populations, resulting in high effective population sizes and high gene flow across populations for the monogyne form. Yet, about 20% of monogyne females mate with polygyne males, and this cross probably yields polygyne colonies (Fontcuberta et al., 2021). Hence, the monogyne and polygyne social forms appear to follow a source–sink dynamics, with asymmetrical gene flow from the monogyne to the polygyne social form (Avril et al., 2019; Ross & Shoemaker, 1997; Seppä et al., 2004). Rare independent colony founding after dispersal flight by polygyne females (Blacher et al., 2021) or by monogyne females mated to polygyne males (Fontcuberta et al., 2021), followed by local budding of polygyne colonies, likely explain the higher inter‐population genetic differentiation in the polygyne social form.

Elevation was a major determinant of genetic structure within regions. First, population differentiation was about six times higher among highland populations than among lowland populations (average *F*_ST_ = 0.058 and 0.01 for highland–highland and lowland–lowland comparisons, respectively). This difference persisted when considering geographical distance: isolation‐by‐distance was significantly stronger among highland populations than among lowland populations. Sample size was small, and pairwise genetic distances were variable, so further research including more highland and lowland population pairs from additional independent valleys will be needed to confirm this pattern. Second, highland populations were genetically less diverse than lowland populations. Restricted gene flow, founder effects and small effective population sizes at high elevations might explain this pattern (Funk et al., 2005; Polato et al., 2017). High ridges of unsuitable habitat separating alpine valleys probably restrict dispersal between highland populations. Founder effects and small effective population sizes are also expected, given harsh climate conditions characterizing high elevation montane habitat (Catalan et al., 2017).

Highland populations are nevertheless connected to nearby lowland populations, as indicated by the lack of effect of elevation distance on genetic differentiation. Gene flow is likely asymmetrical from lowland to highland populations, since strong bidirectional gene flow would homogenize allele frequencies and mask the contrast in connectivity among lowland versus highland populations, respectively. Low genetic diversity and asymmetrical gene flow are consistent with high elevation sites acting as sink populations (Pannell & Charlesworth, 2000; Pulliam, 1988). Overall, our results suggest that gene flow among *F*. *selysi* populations mostly occurs along lowland valleys, and, to a lesser extent, from low to high elevations along secondary steep valleys.

Such pattern of elevated gene flow in lowland areas of mountain regions has been called the “mountain‐valley model” (Funk et al., 2005). It has been found in a variety of montane species, such as chickadees (Branch et al., 2017), frogs (Funk et al., 2005), and mayflies (Polato et al., 2017). Accessible and arable lowland valleys are rare in mountain regions. They are highly exploited for agriculture, industry, roads, and urbanization, which may jeopardize population connectivity of mountain species. Mountains harbor one‐third of the terrestrial biodiversity in the world (Spehn & Körner, 2005) and are a priority for conservation programs (Catalan et al., 2017; CBD, 2010). Yet, protected areas in mountains still fail to cover biodiversity‐important sites (Rodríguez‐Rodríguez et al., 2011). The fact that many montane species rely on lowland riparian corridors for dispersal highlights the need for conserving not only high elevation ecosystems, but also lowland montane habitats.

## CONFLICT OF INTEREST

We declare we have no competing interests.

## AUTHOR CONTRIBUTIONS

**Amaranta Fontcuberta:** Conceptualization (equal); Data curation (equal); Formal analysis (lead); Investigation (lead); Methodology (equal); Visualization (lead); Writing – original draft (lead); Writing – review & editing (lead). **Martin Kapun:** Formal analysis (supporting); Methodology (supporting); Software (supporting); Supervision (supporting). **Patrick Tran Van:** Data curation (supporting); Formal analysis (supporting); Methodology (supporting); Software (supporting). **Jessica Purcell:** Conceptualization (lead); Data curation (equal); Formal analysis (equal); Funding acquisition (supporting); Investigation (supporting); Methodology (supporting); Resources (supporting); Supervision (equal); Validation (equal); Visualization (supporting); Writing – review & editing (supporting). **Michel Chapuisat:** Conceptualization (equal); Formal analysis (supporting); Funding acquisition (lead); Investigation (supporting); Resources (equal); Supervision (lead); Validation (equal); Visualization (supporting); Writing – original draft (supporting); Writing – review & editing (supporting).

## Supporting information

¦ Click here for additional data file.

¦ Click here for additional data file.

¦ Click here for additional data file.

¦ Click here for additional data file.

¦ Click here for additional data file.

¦ Click here for additional data file.

## Data Availability

Single Nucleotide Polymorphism data and R scripts are available from the Dryad digital repository: https://doi.org/10.5061/dryad.hmgqnk9jz. Raw sequence reads have been deposited in NCBI’s sequence read archive under the bioproject PRJNA819254.

## Appendix

**TABLE A1:** Sampling localities, number of colonies sampled (*N*) and individual genotypes at the social supergene

Sampling locality	Region	Lat	Lon	Elevation	*N*	Supergene genotype			Dataset
						*MM*	*MM*	*MM*	
Aubenas (A)	Lower Rhône (FR)	44.6208	4.4220	300	1	0	0	0	1
Buisson (BO)	Lower Rhône (FR)	44.2846	4.9917	180	8	8	8	8	1,2,4
Bussets (BE)	Lower Rhône (FR)	44.2526	5.7188	644	8	5	5	5	1,2,4
St. Michel (SM)	Lower Rhône (FR)	45.2103	6.4812	710	10	10	10	10	1,2
Finges (F)	Upper Rhône (CH)	45.2103	6.4812	565	32	22	22	22	1,2,3,4
Leuk (LK)	Upper Rhône (CH)	46.3121	7.6443	631	14	12	12	12	1,2,3,4
Riddes (R)	Upper Rhône (CH)	46.1786	7.2221	473	4	1	1	1	1,2,3,4
Luette (LU)	Upper Rhône (CH)	46.1583	7.4446	1045	3	0	0	0	1,2,3,4
Les Haudères (H)	Upper Rhône (CH)	46.0821	7.5047	1455	10	7	7	7	1,2,3,4
Derborence (DE)	Upper Rhône (CH)	46.2883	7.2315	1360	27	14	14	14	1,2,3,4
Tamins (T)	Rhine (CH)	46.8137	9.4100	630	18	10	10	10	1,2,3,4
Safien (SF)	Rhine (CH)	46.6835	9.3191	1305	16	16	16	16	1,2,3,4
Dalaas (DA)	Rhine (AT)	47.1270	9.9791	835	1	1	1	1	1
Total					**152**	**106**	**32**	**14**	

Note

FR stands for France, CH for Switzerland, and AT for Austria. Lat = Latitude, Lon = Longitude. *N* = Number of colonies sampled, one worker per colony was genotyped. Dataset 1 was used for Figure 1, Figure 2 and Figure A1; Dataset 2 was used for Figure 3 and Figure A2; Dataset 3 was used for Figure 4 and Figure A3; and Dataset 4 was used for Figure 5.

**TABLE A2:** Environmental variables for isolation by environment analyses

Environmental raster	Resolution	Database	Reference
BIO1 = Annual Mean Temperature	1 km	WorldClim v.1.4 (1)	[1]
BIO2 = Mean Diurnal Range (Mean of monthly (max temp − min temp))	1 km	WorldClim v.1.4 (1)	[1]
BIO3 = Isothermality (BIO2/BIO7) (* 100)	1 km	WorldClim v.1.4 (1)	[1]
BIO4 = Temperature Seasonality (standard deviation *100)	1 km	WorldClim v.1.4 (1)	[1]
BIO5 = Max Temperature of Warmest Month	1 km	WorldClim v.1.4 (1)	[1]
BIO6 = Min Temperature of Coldest Month	1 km	WorldClim v.1.4 (1)	[1]
BIO7 = Temperature Annual Range (BIO5‐BIO6)	1 km	WorldClim v.1.4 (1)	[1]
BIO8 = Mean Temperature of Wettest Quarter	1 km	WorldClim v.1.4 (1)	[1]
BIO9 = Mean Temperature of Driest Quarter	1 km	WorldClim v.1.4 (1)	[1]
BIO10 = Mean Temperature of Warmest Quarter	1 km	WorldClim v.1.4 (1)	[1]
BIO11 = Mean Temperature of Coldest Quarter	1 km	WorldClim v.1.4 (1)	[1]
BIO12 = Annual Precipitation	1 km	WorldClim v.1.4 (1)	[1]
BIO13 = Precipitation of Wettest Month	1 km	WorldClim v.1.4 (1)	[1]
BIO14 = Precipitation of Driest Month	1 km	WorldClim v.1.4 (1)	[1]
BIO15 = Precipitation Seasonality (Coefficient of Variation)	1 km	WorldClim v.1.4 (1)	[1]
BIO16 = Precipitation of Wettest Quarter	1 km	WorldClim v.1.4 (1)	[1]
BIO17 = Precipitation of Driest Quarter	1 km	WorldClim v.1.4 (1)	[1]
BIO18 = Precipitation of Warmest Quarter	1 km	WorldClim v.1.4 (1)	[1]
BIO19 = Precipitation of Coldest Quarter	1 km	WorldClim v.1.4 (1)	[1]
% Bulk density	500 m	LUCAS Topsoil (2)	[2]
% Silt Extra	500 m	LUCAS Topsoil (2)	[2]
% Coarse fragments extra	500 m	LUCAS Topsoil (2)	[2]
% Clay extra	500 m	LUCAS Topsoil (2)	[2]
% Sand extra	500 m	LUCAS Topsoil (2)	[2]
Normalised Difference Vegetation Index (NDVI)*	1 km	MODIS NASA (3)	[3]
Enhanced Vegetation Index (EVI)*	1 km	MODIS NASA (3)	[3]
Elevation (SRTM)	30 m	SRTM, CIAT (4)	[4]

Note

Multivariate “temperature distance” was based on the “Bioclim” variables 1 to 11, “precipitation distance” based on the “Bioclim” variables 12 to 19, “soil distance” based on the five topsoil variables and “vegetation distance” based on two vegetation indexes.

We extracted environmental values from raster data for each population coordinates, using the R package “raster.” *We averaged MODIS rasters for the summer months June, July, August of years 2011 to 2013, to match as close as possible the vegetation during reproductive ant season for the year of sampling (2013) and previous years.
