# Supplementary material for: Effects of social organization and elevation on spatial genetic structure in a montane ant
Source: Ecol Evol. 2022 May 15;12(5):e8813. doi: 10.1002/ece3.8813 (PMC9108227; doi:10.1002/ece3.8813)
Supplement: Supplementary file 4 — ¦ [file ECE3-12-e8813-s001.docx]

**Table S1. Sampling localities, number of colonies sampled (N) and individual genotypes at the social supergene.** FR stands for France, CH for Switzerland, and AT for Austria. Lat = Latitude, Lon = Longitude. N = Number of colonies sampled, one worker per colony was genotyped. Dataset 1 was used for Fig. 1, Fig. 2 and Fig. S1; Dataset 2 was used for Fig. 3 and Fig. S2; Dataset 3 was used for Fig. 4 and Fig. S3; and Dataset 4 was used for Fig. 5.

|  |  |  |  |  |  | **Supergene genotype** | | |  |
| --- | --- | --- | --- | --- | --- | --- | --- | --- | --- |
| **Sampling locality** | **Region** | **Lat** | **Lon** | **Elevation** | **N** | ***MM*** | ***MM*** | ***MM*** | **Dataset** |
| Aubenas (A) | Lower Rhône (FR) | 44.6208 | 4.4220 | 300 | 1 | 0 | 0 | 0 | 1 |
| Buisson (BO) | Lower Rhône (FR) | 44.2846 | 4.9917 | 180 | 8 | 8 | 8 | 8 | 1,2,4 |
| Bussets (BE) | Lower Rhône (FR) | 44.2526 | 5.7188 | 644 | 8 | 5 | 5 | 5 | 1,2,4 |
| St. Michel (SM) | Lower Rhône (FR) | 45.2103 | 6.4812 | 710 | 10 | 10 | 10 | 10 | 1,2 |
| Finges (F) | Upper Rhône (CH) | 45.2103 | 6.4812 | 565 | 32 | 22 | 22 | 22 | 1,2,3,4 |
| Leuk (LK) | Upper Rhône (CH) | 46.3121 | 7.6443 | 631 | 14 | 12 | 12 | 12 | 1,2,3,4 |
| Riddes (R) | Upper Rhône (CH) | 46.1786 | 7.2221 | 473 | 4 | 1 | 1 | 1 | 1,2,3,4 |
| Luette (LU) | Upper Rhône (CH) | 46.1583 | 7.4446 | 1045 | 3 | 0 | 0 | 0 | 1,2,3,4 |
| Les Haudères (H) | Upper Rhône (CH) | 46.0821 | 7.5047 | 1455 | 10 | 7 | 7 | 7 | 1,2,3,4 |
| Derborence (DE) | Upper Rhône (CH) | 46.2883 | 7.2315 | 1360 | 27 | 14 | 14 | 14 | 1,2,3,4 |
| Tamins (T) | Rhine (CH) | 46.8137 | 9.4100 | 630 | 18 | 10 | 10 | 10 | 1,2,3,4 |
| Safien (SF) | Rhine (CH) | 46.6835 | 9.3191 | 1305 | 16 | 16 | 16 | 16 | 1,2,3,4 |
| Dalaas (DA) | Rhine (AT) | 47.1270 | 9.9791 | 835 | 1 | 1 | 1 | 1 | 1 |
| Total | |  |  |  | **152** | **106** | **32** | **14** |  |
