# Supplementary material for: Effects of social organization and elevation on spatial genetic structure in a montane ant
Source: Ecol Evol. 2022 May 15;12(5):e8813. doi: 10.1002/ece3.8813 (PMC9108227; doi:10.1002/ece3.8813)
Supplement: Supplementary file 5 — ¦ [file ECE3-12-e8813-s002.docx]

**Table S2.** **Environmental variables for isolation by environment analyses.** Multivariate “temperature distance” was based on the “Bioclim” variables 1 to 11, “precipitation distance” based on the “Bioclim “variables 12 to 19, “soil distance” based on the five topsoil variables and “vegetation distance” based on two vegetation indexes.

| **Environmental raster** | **Database** | **Resolution** |
| --- | --- | --- |
| BIO1 = Annual Mean Temperature | WorldClim v.1.4 (1) | 1 km |
| BIO2 = Mean Diurnal Range (Mean of monthly (max temp - min temp)) | WorldClim v.1.4 (1) | 1 km |
| BIO3 = Isothermality (BIO2/BIO7) (* 100) | WorldClim v.1.4 (1) | 1 km |
| BIO4 = Temperature Seasonality (standard deviation *100) | WorldClim v.1.4 (1) | 1 km |
| BIO5 = Max Temperature of Warmest Month | WorldClim v.1.4 (1) | 1 km |
| BIO6 = Min Temperature of Coldest Month | WorldClim v.1.4 (1) | 1 km |
| BIO7 = Temperature Annual Range (BIO5-BIO6) | WorldClim v.1.4 (1) | 1 km |
| BIO8 = Mean Temperature of Wettest Quarter | WorldClim v.1.4 (1) | 1 km |
| BIO9 = Mean Temperature of Driest Quarter | WorldClim v.1.4 (1) | 1 km |
| BIO10 = Mean Temperature of Warmest Quarter | WorldClim v.1.4 (1) | 1 km |
| BIO11 = Mean Temperature of Coldest Quarter | WorldClim v.1.4 (1) | 1 km |
| BIO12 = Annual Precipitation | WorldClim v.1.4 (1) | 1 km |
| BIO13 = Precipitation of Wettest Month | WorldClim v.1.4 (1) | 1 km |
| BIO14 = Precipitation of Driest Month | WorldClim v.1.4 (1) | 1 km |
| BIO15 = Precipitation Seasonality (Coefficient of Variation) | WorldClim v.1.4 (1) | 1 km |
| BIO16 = Precipitation of Wettest Quarter | WorldClim v.1.4 (1) | 1 km |
| BIO17 = Precipitation of Driest Quarter | WorldClim v.1.4 (1) | 1 km |
| BIO18 = Precipitation of Warmest Quarter | WorldClim v.1.4 (1) | 1 km |
| BIO19 = Precipitation of Coldest Quarter | WorldClim v.1.4 (1) | 1 km |
| % Bulk density | LUCAS Topsoil (2) | 500 m |
| % Silt Extra | LUCAS Topsoil (2) | 500 m |
| % Coarse fragments extra | LUCAS Topsoil (2) | 500 m |
| % Clay extra | LUCAS Topsoil (2) | 500 m |
| % Sand extra | LUCAS Topsoil (2) | 500 m |
| Normalised Difference Vegetation Index (NDVI) * | MODIS NASA (3) | 1 km |
| Enhanced Vegetation Index (EVI)* | MODIS NASA (3) | 1 km |
| Elevation (SRTM) | SRTM, CIAT (4) | 30m |

We extracted environmental values from raster data for each population coordinates, using the R package “raster”. *We averaged MODIS rasters for the summer months June, July, August of years 2011 to 2013, to match as close as possible the vegetation during reproductive ant season for the year of sampling (2013) and previous years.

References environmental raster data

[1] Hijmans RJ, Cameron SE, Parra JL, Jones PG, Jarvis A. Very high resolution interpolated climate surfaces for global land areas. Int J Climatol. 2005; 25:1965–1978.

[2] Panagos P, Van Liedekerke M, Jones A, Montanarella L. “European Soil Data Centre: Response to European policy support and public data requirements.” Land use policy. 2012;29(2):329–38.

[3] Didan K. MOD13A3 MODIS/Terra vegetation Indices Monthly L3 Global 1km SIN Grid V006. NASA EOSDIS Land Processes DAAC. 2015.

[4] Jarvis A., H.I. Reuter, A. Nelson, E. Guevara, 2008, Hole-filled seamless SRTM data- V4, International Centre for Tropical Agriculture (CIAT), available from <http://srtm.csi.cgiar.org>.
