# Supplementary material for: Effects of social organization and elevation on spatial genetic structure in a montane ant
Source: Ecol Evol. 2022 May 15;12(5):e8813. doi: 10.1002/ece3.8813 (PMC9108227; doi:10.1002/ece3.8813)
Supplement: Supplementary file 6 — ¦ [file ECE3-12-e8813-s006.docx]

# APPENDIX

**Table S1. Sampling localities, number of colonies sampled (N) and individual genotypes at the social supergene.** FR stands for France, CH for Switzerland, and AT for Austria. Lat = Latitude, Lon = Longitude. N = Number of colonies sampled, one worker per colony was genotyped. Dataset 1 was used for Fig. 1, Fig. 2 and Fig. S1; Dataset 2 was used for Fig. 3 and Fig. S2; Dataset 3 was used for Fig. 4 and Fig. S3; and Dataset 4 was used for Fig. 5.

|  |  |  |  |  |  | **Supergene genotype** | | |  |
| --- | --- | --- | --- | --- | --- | --- | --- | --- | --- |
| **Sampling locality** | **Region** | **Lat** | **Lon** | **Elevation** | **N** | ***MM*** | ***MM*** | ***MM*** | **Dataset** |
| Aubenas (A) | Lower Rhône (FR) | 44.6208 | 4.4220 | 300 | 1 | 0 | 0 | 0 | 1 |
| Buisson (BO) | Lower Rhône (FR) | 44.2846 | 4.9917 | 180 | 8 | 8 | 8 | 8 | 1,2,4 |
| Bussets (BE) | Lower Rhône (FR) | 44.2526 | 5.7188 | 644 | 8 | 5 | 5 | 5 | 1,2,4 |
| St. Michel (SM) | Lower Rhône (FR) | 45.2103 | 6.4812 | 710 | 10 | 10 | 10 | 10 | 1,2 |
| Finges (F) | Upper Rhône (CH) | 45.2103 | 6.4812 | 565 | 32 | 22 | 22 | 22 | 1,2,3,4 |
| Leuk (LK) | Upper Rhône (CH) | 46.3121 | 7.6443 | 631 | 14 | 12 | 12 | 12 | 1,2,3,4 |
| Riddes (R) | Upper Rhône (CH) | 46.1786 | 7.2221 | 473 | 4 | 1 | 1 | 1 | 1,2,3,4 |
| Luette (LU) | Upper Rhône (CH) | 46.1583 | 7.4446 | 1045 | 3 | 0 | 0 | 0 | 1,2,3,4 |
| Les Haudères (H) | Upper Rhône (CH) | 46.0821 | 7.5047 | 1455 | 10 | 7 | 7 | 7 | 1,2,3,4 |
| Derborence (DE) | Upper Rhône (CH) | 46.2883 | 7.2315 | 1360 | 27 | 14 | 14 | 14 | 1,2,3,4 |
| Tamins (T) | Rhine (CH) | 46.8137 | 9.4100 | 630 | 18 | 10 | 10 | 10 | 1,2,3,4 |
| Safien (SF) | Rhine (CH) | 46.6835 | 9.3191 | 1305 | 16 | 16 | 16 | 16 | 1,2,3,4 |
| Dalaas (DA) | Rhine (AT) | 47.1270 | 9.9791 | 835 | 1 | 1 | 1 | 1 | 1 |
| Total | |  |  |  | **152** | **106** | **32** | **14** |  |

**Table S2.** **Environmental variables for isolation by environment analyses.** Multivariate “temperature distance” was based on the “Bioclim” variables 1 to 11, “precipitation distance” based on the “Bioclim “variables 12 to 19, “soil distance” based on the five topsoil variables and “vegetation distance” based on two vegetation indexes.

| **Environmental raster** | **Database** | **Resolution** |
| --- | --- | --- |
| BIO1 = Annual Mean Temperature | WorldClim v.1.4 (1) | 1 km |
| BIO2 = Mean Diurnal Range (Mean of monthly (max temp - min temp)) | WorldClim v.1.4 (1) | 1 km |
| BIO3 = Isothermality (BIO2/BIO7) (* 100) | WorldClim v.1.4 (1) | 1 km |
| BIO4 = Temperature Seasonality (standard deviation *100) | WorldClim v.1.4 (1) | 1 km |
| BIO5 = Max Temperature of Warmest Month | WorldClim v.1.4 (1) | 1 km |
| BIO6 = Min Temperature of Coldest Month | WorldClim v.1.4 (1) | 1 km |
| BIO7 = Temperature Annual Range (BIO5-BIO6) | WorldClim v.1.4 (1) | 1 km |
| BIO8 = Mean Temperature of Wettest Quarter | WorldClim v.1.4 (1) | 1 km |
| BIO9 = Mean Temperature of Driest Quarter | WorldClim v.1.4 (1) | 1 km |
| BIO10 = Mean Temperature of Warmest Quarter | WorldClim v.1.4 (1) | 1 km |
| BIO11 = Mean Temperature of Coldest Quarter | WorldClim v.1.4 (1) | 1 km |
| BIO12 = Annual Precipitation | WorldClim v.1.4 (1) | 1 km |
| BIO13 = Precipitation of Wettest Month | WorldClim v.1.4 (1) | 1 km |
| BIO14 = Precipitation of Driest Month | WorldClim v.1.4 (1) | 1 km |
| BIO15 = Precipitation Seasonality (Coefficient of Variation) | WorldClim v.1.4 (1) | 1 km |
| BIO16 = Precipitation of Wettest Quarter | WorldClim v.1.4 (1) | 1 km |
| BIO17 = Precipitation of Driest Quarter | WorldClim v.1.4 (1) | 1 km |
| BIO18 = Precipitation of Warmest Quarter | WorldClim v.1.4 (1) | 1 km |
| BIO19 = Precipitation of Coldest Quarter | WorldClim v.1.4 (1) | 1 km |
| % Bulk density | LUCAS Topsoil (2) | 500 m |
| % Silt Extra | LUCAS Topsoil (2) | 500 m |
| % Coarse fragments extra | LUCAS Topsoil (2) | 500 m |
| % Clay extra | LUCAS Topsoil (2) | 500 m |
| % Sand extra | LUCAS Topsoil (2) | 500 m |
| Normalised Difference Vegetation Index (NDVI) * | MODIS NASA (3) | 1 km |
| Enhanced Vegetation Index (EVI)* | MODIS NASA (3) | 1 km |
| Elevation (SRTM) | SRTM, CIAT (4) | 30m |

We extracted environmental values from raster data for each population coordinates, using the R package “raster”. *We averaged MODIS rasters for the summer months June, July, August of years 2011 to 2013, to match as close as possible the vegetation during reproductive ant season for the year of sampling (2013) and previous years.

References environmental raster data

[1] Hijmans RJ, Cameron SE, Parra JL, Jones PG, Jarvis A. Very high resolution interpolated climate surfaces for global land areas. Int J Climatol. 2005; 25:1965–1978.

[2] Panagos P, Van Liedekerke M, Jones A, Montanarella L. “European Soil Data Centre: Response to European policy support and public data requirements.” Land use policy. 2012;29(2):329–38.

[3] Didan K. MOD13A3 MODIS/Terra vegetation Indices Monthly L3 Global 1km SIN Grid V006. NASA EOSDIS Land Processes DAAC. 2015.

[4] Jarvis A., H.I. Reuter, A. Nelson, E. Guevara, 2008, Hole-filled seamless SRTM data- V4, International Centre for Tropical Agriculture (CIAT), available from <http://srtm.csi.cgiar.org>.

**Figure S1. Determination of the social supergene genotype.** X-axis displays the first component (PC1) of a PCA of SNPs on chromosome 3, which contains the social supergene. Y-axis represents the *F*_IS_ per individual: negative values indicate heterozygous genotypes and positive values indicate homozygous genotypes. The three groups correspond to the social supergene genotypes *PP* (red), *PM* (orange) and *MM* (blue), respectively. *PP* and *PM* individuals belong to the polygyne social form, whereas *MM* individuals belong to the monogyne social form (Purcell et al. 2014; Avril et al. 2019).

**
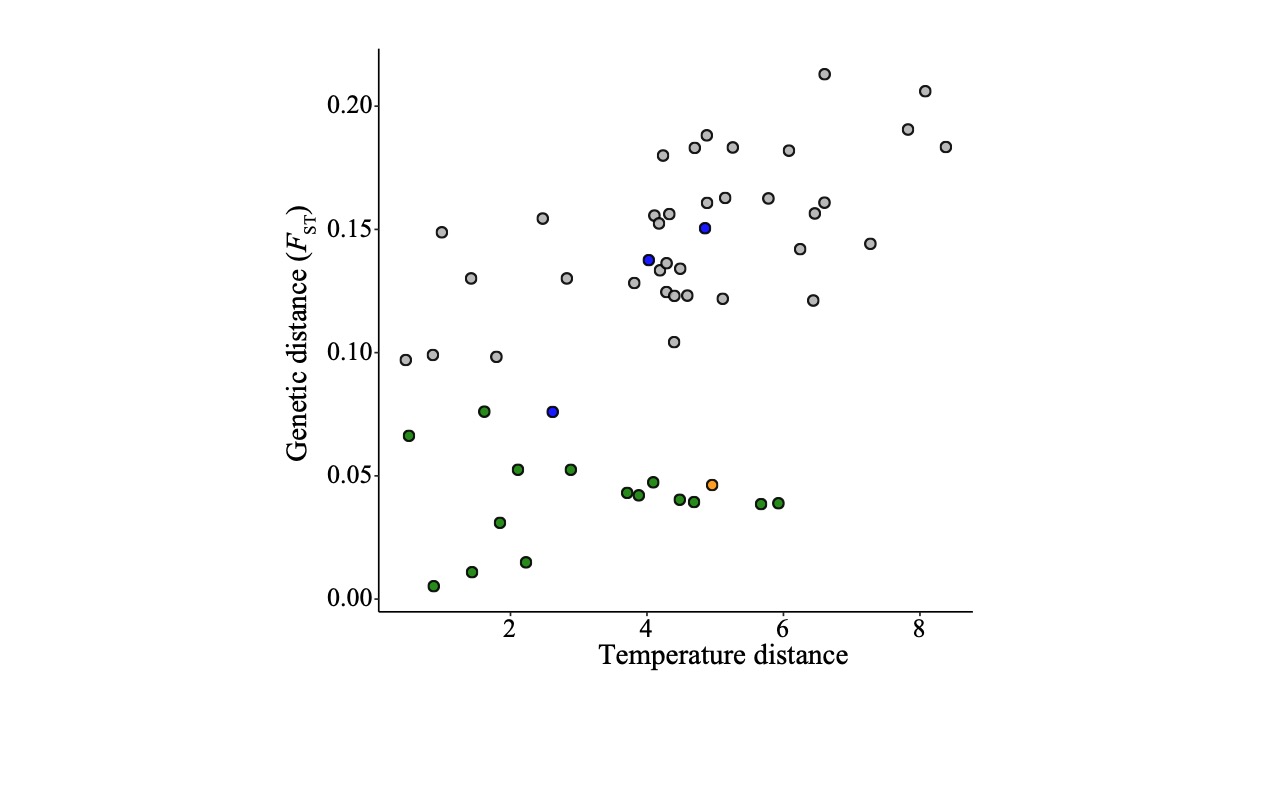
**

**Figure S2. Isolation by environment.** Relation between genetic distance (*F*_ST_) and multivariate temperature distance. Coloured dots are population pairs within regions (blue: Lower Rhône, green: Upper Rhône, orange: Rhine), and grey dots represent population pairs from different regions. Includes populations BO, BE and SM in Lower Rhône, all populations in Upper Rhône, T and S populations in Rhine region (Table S1).

**Figure S3. Genetic diversity in lowland (black) and highland (white) populations**. Each dot represents a population (circles: Upper Rhône, triangles: Rhine). Includes all populations in Upper Rhône region, and populations T and S in Rhine region (Table S1).
